# Unusual Manifestations of Sinonasal Osteomas: A Narrative Review

**DOI:** 10.3390/clinpract16070126

**Published:** 2026-07-04

**Authors:** Spyridon Lygeros, Alkmini Gatsounia, Ioanna Athanasiadou, Aris I. Giotakis, Foteini Tsapardoni, Gerasimos Danielides

**Affiliations:** 1 Department of Otorhinolaryngology, University of Patras, 26504 Patras, Greece; alkgats@gmail.com (A.G.); ioannatha333@outlook.com.gr (I.A.); 3idani23@gmail.com (G.D.); 2 First Department of Otorhinolaryngology, Head and Neck Surgery, National and Kapodistrian University of Athens, Hippocrateion General Hospital, 11527 Athens, Greece; arisgiotakis@gmail.com; 3 Department of Ophthalmology, University Hospital of Patras, 26504 Patras, Greece; fentsapardoni@gmail.com

**Keywords:** osteoma, paranasal sinus neoplasms, pneumocephalus, mucocele, orbital diseases, skull base

## Abstract

**Background/Objectives**: Sinonasal osteomas are benign, slow-growing tumors that are typically asymptomatic and incidentally detected. However, in rare cases, they may present with atypical and potentially serious complications involving other sinonasal, orbital, or intracranial structures. This review aims to synthesize these unusual manifestations and to highlight the underlying mechanisms, diagnostic challenges, and management implications. **Methods**: A narrative review of the literature was conducted, focusing on reported cases of sinonasal osteomas with rare or complicated presentations. Studies were analyzed with emphasis on clinical features, imaging findings, pathophysiological mechanisms, and treatment strategies. **Results**: Unusual presentations of sinonasal osteomas are primarily driven by sinus obstruction, progressive expansion, and skull-base erosion. These processes may result in complications such as pneumocephalus, intracranial mucoceles, cerebrospinal fluid leaks, orbital compression, and secondary infections. Clinical manifestations are often nonspecific, including headache, seizures, visual disturbances, or focal neurological deficits, which may delay diagnosis. High-resolution computed tomography is essential for identifying the osseous lesion and associated bone changes, while magnetic resonance imaging is critical for assessing soft-tissue involvement and intracranial extension. Management is individualized, with surgical resection indicated in most symptomatic or complicated cases, using endoscopic, open, or combined approaches. **Conclusions**: Although rare, atypical manifestations of sinonasal osteomas can result in significant morbidity. A mechanism-based understanding, supported by appropriate imaging, is essential for accurate diagnosis and timely management. Increased clinical awareness is crucial to improving outcomes in these uncommon but clinically significant cases.

## 1. Introduction

Paranasal sinus osteomas are benign, slow-growing osseous neoplasms and represent the most common fibro-osseous tumors of the sinonasal region. They are typically discovered incidentally on imaging, with a prevalence of approximately 3% in the general population [1]. Although their exact etiology remains uncertain, aberrant osteoblastic activity—potentially triggered by developmental anomalies, trauma, or chronic inflammation—appears responsible [2]. The frontal sinus is most frequently involved (52–80%), followed by the ethmoid (20–30%), while maxillary and sphenoid lesions are uncommon [3].

The pathophysiology of sinonasal osteoma formation remains incompletely understood and is likely multifactorial. Several mechanisms have been proposed, including developmental, traumatic, inflammatory, and genetic theories. The developmental theory suggests that osteomas may arise from embryologic remnants or abnormal bone maturation within pneumatized sinonasal structures, whereas the traumatic theory implicates micro-injury or prior surgery as potential triggers of reactive osteogenesis [1,4]. Chronic inflammation may also contribute by stimulating persistent osteoblastic activity and localized bone remodeling, resulting in aberrant bone growth [5]. A genetic predisposition is supported by Gardner’s syndrome, in which multiple osteomas occur in association with intestinal polyposis [6]. Although these mechanisms may explain osteoma formation, the unusual manifestations reviewed in this article are more closely related to secondary processes such as sinus outflow obstruction, progressive bony expansion, and erosion of adjacent structures [4].

Osteomas are classified by histology into ivory (dense, compact bone), mature (cancellous with marrow spaces), or mixed types [7]. Growth is usually indolent (0.3–1 mm/year), with many lesions remaining stable for decades. However, rare cases exhibit accelerated enlargement, resulting in extensive bony expansion and mass effect—as described in a giant intracranial mucocele complicating a frontal sinus osteoma [8].

Clinical presentation depends predominantly on tumor size and location. Due to their slow growth, only a minority of sinonasal osteomas become symptomatic, with larger series reporting approximately 10% [9]. Small, intrasinus osteomas are often asymptomatic and discovered on CT. Larger lesions may obstruct sinus outflow, impair mucociliary clearance, and cause nasal congestion, postnasal drip, recurrent sinusitis, or chronic headache [10]. Frontal osteomas typically present with localized headache, while ethmoid lesions can compress orbital structures, leading to diplopia, epiphora, or proptosis. Maxillary osteomas may produce facial pain, swelling, or dental issues, and sphenoid osteomas—though rare—can cause retro-orbital pain or visual disturbances [11]. Tumor expansion beyond the sinus walls, although uncommon, further raises the risk of intracranial or orbital extension, mucocele formation, and secondary infections.

Early recognition of these unusual presentations is crucial to prevent severe morbidity. The often-nonspecific symptoms—headache, cognitive changes, diplopia, or proptosis—may be misattributed to primary neurologic or orbital conditions. For example, pneumocephalus with a CSF leak can be mistaken for a primary intracranial infection or idiopathic headache syndrome, and orbital invasion may mimic neoplastic or inflammatory orbital disease, delaying definitive treatment [7,12]. Moreover, dural erosion can permit mucopyocele, orbital cellulitis, or intracranial abscess formation—complications linked to permanent vision loss and neurologic deficits when diagnosis is delayed [4,13].

This review therefore consolidates the literature on these uncommon and complex osteoma cases, examines the diagnostic challenges posed by their deceptive presentations, and evaluates management strategies—from conservative monitoring to endoscopic and open surgical techniques. By highlighting these rare but consequential complications, we aim to improve early detection, refine surgical planning, and ultimately enhance patient outcomes.

## 2. Methods

A narrative review of the literature was conducted to identify reports describing unusual manifestations, uncommon anatomical locations, and rare complications of sinonasal osteomas. The literature search was performed using the PubMed database and was supplemented by manual screening of the reference lists of relevant articles to identify additional publications.

The search strategy included combinations of keywords such as “sinonasal osteoma”, “paranasal sinus osteoma”, “frontal sinus osteoma”, “ethmoid osteoma”, “sphenoid sinus osteoma”, “orbital complications”, “pneumocephalus”, “mucocele”, “cerebrospinal fluid leak”, “meningitis”, “brain abscess”, and “visual impairment”.

Eligible studies included case reports, case series, and review articles describing atypical clinical manifestations, uncommon anatomical locations, or neurological, orbital, infectious, pneumocephalus-related, and mucocele-associated complications of sinonasal osteomas. Preference was given to studies providing detailed clinical, radiological, diagnostic, therapeutic, and outcome data, as well as to more recent publications when multiple reports described similar presentations. Due to the number and heterogeneity of published reports, the tables included in this review summarize representative cases selected for their clinical relevance, unusual presentation, and completeness of diagnostic and management information rather than all cases identified during the literature search.

Articles lacking sufficient clinical information or focusing exclusively on uncomplicated osteomas without unusual manifestations were not included. Due to the rarity and heterogeneity of these presentations, the available evidence was synthesized narratively, with emphasis on clinical presentation, diagnostic evaluation, imaging findings, management strategies, and the spectrum of reported complications.

## 3. Rare Presentations of Sinonasal Osteomas

### 3.1. Pneumocephalus

Pneumocephalus results from a discontinuity of the skull base that allows air to enter the cranial cavity. While most commonly associated with trauma, infection, or iatrogenic causes, paranasal osteomas may rarely erode the posterior sinus wall and dura, creating a communication that permits intracranial air accumulation. Clinically, patients may present with headache, seizures, focal neurological deficits, hemiparesis, cognitive impairment, personality changes, or language disturbances, depending on the location and extent of intracranial air accumulation [14,15,16,17,18]. This complication was first described by Cushing in 1927 [19]. In severe cases, tension pneumocephalus may cause signs of increased intracranial pressure, including nausea, vomiting, altered consciousness, and cerebrospinal fluid rhinorrhea, representing a neurosurgical emergency [20].

Two mechanisms have been proposed to explain osteoma-related pneumocephalus. The “inverted-bottle” mechanism involves continuous cerebrospinal fluid leakage through a dural defect, generating negative intracranial pressure that draws air intracranially, whereas the “ball-valve” mechanism occurs when transient increases in sinus pressure force air through a skull-base defect and subsequently trap it within the cranial cavity [16,21].

High-resolution CT and MRI are useful for identifying the osteoma, associated skull-base defects, intracranial air collections, and adjacent soft-tissue involvement [19]. Reported treatment strategies generally include decompression of trapped air, osteoma removal, sinus management, and dural repair through endoscopic, open, or combined approaches, depending on lesion size and extent [17,19,21,22].

Large osteomas have been associated with complex intracranial complications, including combined pneumocephalus and mucocele formation, particularly in the presence of skull-base erosion [20]. Representative cases of osteoma-related pneumocephalus are summarized in Table 1.

### 3.2. Mucocele Formation

Among reported complicated osteoma cases, secondary mucoceles are a frequent finding, although intracranial extension remains uncommon [28,29]. When an osteoma obstructs a sinus ostium, mucus produced by the respiratory mucosa accumulates, forming a mucocele—an epithelial-lined, mucus-filled cystic cavity. Progressive expansion increases intracystic pressure, leading to bony erosion and, in some cases, establishment of a one-way valve mechanism that entraps air [4,28].

Neurological manifestations are particularly common in cases of intracranial mucocele formation. Reported presentations include seizures, frontal lobe syndrome, cognitive decline, personality changes, and focal neurological deficits resulting from direct compression of adjacent brain structures [4,28,30,31].

Clinically, headache is commonly reported in cases of intracranial mucocele secondary to osteoma [4,28]. Visual disturbances—such as diplopia, blepharoptosis, and reduced acuity—may occur when lesions approach the orbit [4,29]. Neurological manifestations range from seizures and personality changes to focal motor or sensory deficits [29,31]. A superimposed infection can convert a mucocele into a mucopyocele, raising the risk of meningitis, abscess formation, or cerebrospinal fluid fistula [13,28].

Intracranial extension remains rare. Licci et al. identified 26 reported cases, with only two complicated by abscess, while Sakamoto et al. reported 21 cases of coexisting osteoma and intracranial mucocele. Nabeshima et al. described eleven histopathologically confirmed cases, most involving extension into the frontal lobe. Such cases may also present with pneumocephalus when dural defects permit air entry [4,28].

Representative cases of osteoma-associated mucoceles and their complications are summarized in Table 2.

### 3.3. Infectious Complications

Infections associated with paranasal osteomas may extend beyond the sinonasal cavity and involve adjacent structures, most notably the central nervous system and the orbit. Reported complications include meningitis, subdural empyema, intracerebral abscesses, orbital cellulitis, and frontal osteomyelitis. Obstruction of the sinus ostium may create a closed environment that favors bacterial proliferation, while erosion of the posterior sinus wall can facilitate direct intracranial spread of infection. These cases emphasize the potentially severe consequences of delayed diagnosis and treatment [13,35,36].

Several notable cases highlight the infectious complications linked to osteoma-related pathology. In one case, a 51-year-old man developed seizures, aphasia, and hemiparesis due to a frontal lobe abscess caused by a frontal sinus osteoma; Streptococcus pneumoniae was isolated, and combined craniotomy, abscess drainage, and tumor resection led to full recovery [37]. Another report described a 38-year-old man with a long-standing history of seizures and headaches who developed bacterial meningitis from a giant frontal osteoma with dural erosion. Imaging revealed an intraparenchymal abscess, necessitating joint neurosurgical and ENT resection [6].

Infections can also result from mucocele transformation. A 71-year-old man with a recurrent osteoma presented with confusion and fever due to a mucopyocele and intracranial abscess caused by Moraxella catarrhalis—a rare pathogen in this context [13]. Another case involved a 34-year-old man with a giant frontoethmoidal osteoma eroding the sinus wall and dura, resulting in subdural empyema and midline shift. Emergency craniotomy and osteoma resection were lifesaving [38]. A 15-year-old boy developed Pott’s puffy tumor—frontal osteomyelitis with subperiosteal abscess—due to an undiagnosed frontal osteoma that eroded the anterior sinus wall [36].

Orbital infections have also been documented. A 30-year-old woman experienced acute orbital cellulitis with proptosis due to an ethmoidal osteoma breaching the orbit; combined endoscopic and external surgery led to a good outcome [35]. In an unusual case, a 17-year-old girl with allergic rhinitis was found to have a nasal septal osteoma colonized by Actinomyces species, attributed to localized hypoxia from septal narrowing; endoscopic removal and short-course penicillin resolved the infection [39]. Representative cases with infectious complications secondary to sinonasal osteomas are summarized in Table 3. 

### 3.4. Orbital Manifestations

Paranasal sinus osteomas with orbital involvement are uncommon but clinically significant, as they may cause proptosis, diplopia, ptosis, optic neuropathy, and visual loss. These manifestations arise when osteomas originating from the frontal, ethmoid, or sphenoid sinuses extend into the orbit or optic canal, compressing adjacent structures.

Clinical presentation varies according to tumor size, location, and growth rate. In a notable case, a 14-year-old boy with a sphenoethmoidal osteoma developed unilateral visual loss and optic atrophy due to optic nerve compression; surgical decompression resulted in visual improvement [40]. Handa et al. reported a 28-year-old man with a giant frontoethmoidal osteoma causing significant globe displacement without visual impairment, successfully managed with a combined approach [7]. Less severe manifestations, such as ptosis and orbital swelling, have also been described and treated effectively with limited surgical approaches [41].

Larger lesions are more likely to demonstrate intraorbital extension and mass effect. In a clinical series, proptosis and diplopia were the most frequent findings, with some patients developing visual impairment secondary to optic nerve compression [42]. A summary of reported orbital complications associated with sinonasal osteomas is provided in Table 4.

### 3.5. Osteomas in Rare Anatomical Locations

Although sinonasal osteomas most commonly arise within the frontal and ethmoid sinuses, rare cases have been reported in atypical anatomical locations, including the middle turbinate (concha bullosa), inferior turbinate, nasal septum, nasal bones, and sphenoid sinus. Due to their concealed location and often nonspecific symptomatology, these lesions may present with isolated headache, facial swelling, or nasal obstruction, frequently in the absence of remarkable endoscopic findings. In such cases, computed tomography remains essential for accurate diagnosis and preoperative planning.

Several unusual locations have been described in the literature. A middle concha bullosa osteoma presenting with intractable frontal headache was successfully managed with endoscopic removal [43]. Similarly, an osteoma arising within a pneumatized nasal septum caused chronic frontal headache and was successfully treated endoscopically [44]. An inferior turbinate osteoma has also been reported, presenting with nasal obstruction, facial pain, and headache [45]. Rare osteomas involving the nasal bones typically present as slowly enlarging facial swellings and have been successfully managed surgically [46,47]. Sphenoid sinus osteomas deserve particular attention because of their proximity to critical neurovascular structures. A large sphenoid sinus osteoma presenting with progressive headache was reported in a young woman and successfully treated through an endoscopic approach [48].

Collectively, these reports highlight the diagnostic challenges posed by osteomas arising in uncommon anatomical locations. Because symptoms are often nonspecific and endoscopic findings may be unremarkable, diagnosis may be delayed, emphasizing the importance of computed tomography in patients with persistent rhinologic or facial symptoms. A summary of these cases is presented in Table 5.

## 4. Discussion

Although paranasal sinus osteomas are generally regarded as benign and indolent lesions, the cases reviewed above demonstrate that they may occasionally present with clinically significant and potentially life-threatening complications. These atypical manifestations appear to arise when specific anatomical and functional thresholds are exceeded—most notably sinus obstruction and progressive bone erosion.

Mechanical obstruction and pressure-related dynamics appear to underlie most of the reported complications. Obstruction of the sinus ostium—particularly at the frontonasal duct—promotes mucus retention and subsequent mucocele formation, which may progressively expand and lead to bony remodeling, erosion, and eventual intracranial or orbital extension [4,28]. Concurrently, erosion of the posterior sinus wall and dura establishes a pathological communication that allows the development of pneumocephalus through mechanisms such as the “ball-valve” and “inverted-bottle” phenomena [19,21]. In clinical practice, these processes often coexist rather than occur in isolation, explaining the overlap between neurological, infectious, and orbital presentations.

From an anatomical perspective, complicated cases are most frequently associated with frontal and ethmoidal osteomas, reflecting both their higher baseline prevalence and their proximity to the orbit and anterior cranial fossa [3]. Their predominance among reported complications should therefore be interpreted cautiously, as it may be influenced by frequency rather than an inherently more aggressive biological behavior. Nevertheless, their anatomical relationships account for the diversity of clinical manifestations, including orbital symptoms and neurological involvement [16,32].

An important observation is that neurological symptoms represent a common clinical consequence of several underlying pathological processes. Headache, seizures, hemiparesis, cognitive decline, and behavioral changes were reported across cases of pneumocephalus, intracranial mucocele formation, infection, and extensive orbital or intracranial extension. Consequently, neurological manifestations should be interpreted within the context of the underlying complication rather than as a separate disease entity.

### 4.1. Diagnostic Challenges

A consistent finding across the reported cases is that clinical presentation is often nonspecific, frequently leading to delayed diagnosis. Symptoms such as headache, seizures, visual disturbances, focal neurological deficits, or cerebrospinal fluid rhinorrhea may initially be attributed to more common neurological, ophthalmological, or infectious conditions, particularly in the absence of obvious sinonasal findings. This challenge is further amplified when osteomas arise in uncommon anatomical locations such as the concha bullosa, nasal septum, inferior turbinate, or sphenoid sinus, where endoscopic findings may be limited or absent.

#### 4.1.1. Differential Diagnosis

The differential diagnosis of atypically presenting sinonasal osteomas includes a variety of benign, infectious, and malignant lesions. Among benign fibro-osseous tumors, fibrous dysplasia, ossifying fibroma, and osteoblastoma may demonstrate overlapping radiological features, although they typically differ in their patterns of growth, internal architecture, and bone remodeling [1,49]. In particular, osteomas with osteoblastoma-like features may exhibit expansile behavior, atypical imaging characteristics, and the characteristic “sclerotic cap” sign, which may assist in differentiating them from other fibro-osseous lesions [49]. Chronic fungal sinusitis may also present as a hyperdense calcified sinonasal lesion and should be considered in the appropriate clinical setting. Malignant neoplasms involving the sinonasal tract, including osteosarcoma, chondrosarcoma, and sinonasal undifferentiated carcinoma, are generally characterized by more aggressive bone destruction, soft tissue invasion, and rapid progression compared with the indolent growth pattern of osteomas [1].

#### 4.1.2. Diagnostic Workup and the Role of Imaging

The diagnostic workup of patients with suspected complicated sinonasal osteomas should be guided by the presenting symptoms and the suspected complication. Because these complications may initially manifest with neurological, ophthalmological, or infectious symptoms, patients are frequently evaluated by neurologists, ophthalmologists, or emergency physicians before referral to an otolaryngologist. In many reported cases, cross-sectional imaging had already been performed before the underlying sinonasal pathology was recognized.

Following clinical suspicion, a complete otolaryngological evaluation should include nasal endoscopy as the initial examination. Although endoscopic findings may be limited or even normal, particularly in osteomas arising from uncommon anatomical locations, nasal endoscopy remains essential for identifying associated mucosal abnormalities, obstruction of the sinus drainage pathways, or evidence of cerebrospinal fluid leakage. Patients presenting with neurological manifestations should also undergo a thorough neurological examination, while ophthalmologic assessment is recommended in cases of visual symptoms, proptosis, diplopia, or suspected optic canal involvement.

Imaging plays a central role in both diagnosis and treatment planning. High-resolution computed tomography (CT) with multiplanar reconstruction remains the gold standard for evaluating sinonasal osteomas, accurately defining lesion size, density, anatomical extent, bony remodeling, skull-base defects, and orbital or intracranial extension [9,29]. CT is also indispensable for preoperative planning and, when available, image-guided surgical navigation. Cone-beam CT may provide high-resolution assessment of osseous structures with reduced radiation exposure in selected cases.

Magnetic resonance imaging (MRI) serves a complementary role by providing superior evaluation of soft-tissue involvement, dural integrity, orbital extension, intracranial complications, abscess formation, edema, and the cystic content of secondary mucoceles [21,29,31]. Contrast-enhanced MRI is particularly useful when intracranial or orbital extension is suspected and plays an important role in the differential diagnosis of lesions with significant soft-tissue components.

Additional investigations should be performed selectively according to the clinical scenario. When cerebrospinal fluid rhinorrhea is suspected, β-2 transferrin or β-trace protein testing may help confirm the presence of CSF, whereas CT cisternography should generally be reserved for selected patients in whom localization of the leak remains uncertain despite conventional imaging [32]. In patients with suspected sinonasal infection, appropriate microbiological sampling, including nasal or sinus cultures when feasible, may help guide antimicrobial therapy [13]. Blood cultures should be obtained in patients with evidence of systemic infection, while lumbar puncture with cerebrospinal fluid analysis should be performed when meningitis is clinically suspected, and there are no contraindications [6]. These investigations should always be interpreted in conjunction with the clinical presentation and imaging findings.

### 4.2. Management Implications

Management of atypically presenting sinonasal osteomas depends on symptom severity, anatomical location, lesion extent, and the presence of associated complications. While asymptomatic lesions may be managed conservatively with periodic radiological surveillance, surgical treatment is generally indicated for symptomatic tumors, progressive growth, obstruction of the nasofrontal duct, significant frontal sinus involvement, skull-base erosion, orbital compression, or intracranial extension [9,25].

The choice of surgical approach is primarily determined by tumor location and extent. Endoscopic sinus surgery has become increasingly favored for appropriately selected lesions, particularly those confined to the ethmoid sinus, frontal recess, or other accessible locations, offering effective treatment with reduced morbidity and faster recovery. However, open or combined approaches remain necessary for giant osteomas and for cases involving the orbit, optic canal, anterior skull base, extensive frontal sinus disease, or intracranial complications [4,19,20,33].

The management of complicated osteomas frequently requires treatment of both the lesion itself and its secondary consequences. Reported cases of pneumocephalus often required decompression of trapped intracranial air, osteoma removal, sinus management, and dural reconstruction using autologous grafts such as fascia lata or pericranium [22]. In patients undergoing surgical repair of cerebrospinal fluid fistulas, intrathecal fluorescein may facilitate intraoperative localization of the leak and assist in confirming successful closure of the skull-base defect [11]. Similarly, osteomas associated with mucoceles may require complete resection of both lesions, restoration of sinus drainage, and, in selected cases, cranialization or obliteration procedures [4,28,33]. Infectious complications, including meningitis, intracranial abscesses, and empyema, often necessitate multidisciplinary management combining surgery with culture-guided antimicrobial therapy, while orbital involvement may require early decompression to preserve visual function [13,35].

Because these atypical presentations frequently involve the skull base, orbit, or intracranial compartment, management often requires close collaboration among otolaryngologists, neurosurgeons, ophthalmologists, and infectious disease specialists. Reconstruction of skull-base and orbital defects may be necessary in selected patients to restore function and prevent long-term complications. Overall, postoperative outcomes are generally favorable when complete resection and appropriate reconstruction are achieved, although careful long-term follow-up remains essential to identify recurrence or delayed complications.

## 5. Conclusions

Although most paranasal sinus osteomas are benign and asymptomatic, rare cases can lead to serious complications involving the orbit, brain, or surrounding tissues. These unusual presentations are often misdiagnosed, making early recognition and appropriate imaging essential. Management should be tailored to each case, with observation reserved for stable lesions and surgery considered when there is a risk of functional or structural damage. Advances in endoscopic techniques have improved outcomes, although complex cases may still require open or combined approaches. Given that the available evidence is largely derived from case reports and small case series, a high degree of clinical suspicion remains necessary when evaluating atypical presentations. Ultimately, a multidisciplinary approach and greater awareness of these rare manifestations are key to timely diagnosis, appropriate treatment, and improved patient outcomes.

## Figures and Tables

**Table 1:** Summary of reported cases of pneumocephalus complicating paranasal sinus osteomas.

Study	Age (Years), Sex & Presentation	Imaging Findings	Pneumocephalus Location/Mechanism	Management and Outcome
Wu et al. 2011 [14]	14/M: acute left hemiparesis and ataxia after trumpet playing	Ethmoidal osteoma breaching dura with large frontal tension pneumatocele	Ball-valve: air forced through dural defect and trapped	Craniotomy, complete excision, dural repair → full recovery
Umredkar et al. 2017 [15]	22/M: 2 yrs intermittent headache, then 1 month progressive left weakness	Frontal sinus osteoma eroding posterior wall, extradural air–fluid collection	Ball-valve: positive-pressure air entry via dural breach	Surgical excision and dural repair → full recovery
Pathak et al. 2020 [16]	45/M: progressive apathy, disinhibition, and memory loss consistent with frontotemporal dementia	Ethmoidal osteoma with dural breach and bilateral frontal intraparenchymal pneumocephalus (“Mount-Fuji” sign)	Ball-valve/inverted-bottle: air entry through dural defect or CSF egress creating negative pressure	Surgery planned, but patient died of sudden cardiac arrest before intervention
Mahabir et al. (2004) [19]	68/M: sudden severe headache, confusion and vomiting during flight; 2 d delay and frontal lobe syndrome (disorientation, behavioral changes).	CT: 5.6 × 5.5 × 6.5 cm right frontal intraparenchymal air collection and ethmoid osteoma; MRI: hypointense calcified ethmoid mass and 1.3 cm midline shift.	Intraparenchymal (frontal)	Bicoronal craniotomy with intracranial osteoma resection, periosteal/dural repair and partial ethmoid removal; postoperative CT on Day 2 showed re-expansion, and at 6 mo there was no recurrence.
Johnson & Tan (2002) [23]	62/M: 2-week vague personality changes progressing to drowsiness, headache, nausea and left hemiparesis.	CT: right frontal subcortical pneumatocele under cortex with frontal sinus osteoma breaching dura; intraoperative air bubbles confirmed intraparenchymal tension air.	Intraparenchymal (subcortical)	Right frontal craniotomy, partial osteoma removal, frontal sinus mucosa debridement and direct dural suture repair; uneventful recovery, discharged Day 10, CT at 3 mo showed complete resolution.
Harasaki et al. (2013) [12]	61/F: 2-month progressive right hemiparesis and ataxia (prior frontal trauma 18 mo earlier).	CT: frontoethmoid osteoma through cribriform plate with intracranial air tracking into parenchyma; MRI: pneumatocele dissecting into the left frontal subcortical white matter.	Intraparenchymal (left frontal)	(1) Frameless stereotactic burr-hole decompression of the air collection; (2) endoscopic Draf III resection of osteoma and septal mucosal graft dural repair. Strength fully restored by POD 1; near-total resolution on 6 mo MRI.
Nakayama et al. (1998) [24]	35/F: sudden severe headache triggered by nose-blowing and right-sided anosmia; no meningeal signs.	CT: right ethmoid osteoma with skull-base and dural defect, multiple air bubbles in interhemispheric, ambient, interpeduncular and sylvian cisterns.	Cisternal (subarachnoid)	Wide bilateral frontal craniotomy, extradural elevation of frontal lobe, drilling/removal of intracranial component, and fascia graft dural repair; complete resolution of pneumocephalus, uneventful recovery.
Keshri et al. (2025) [25]	33/M: 20 days tension frontal headache, progressive right-sided hemiparesis	CT: lobulated dense mass in ethmoid and left frontal sinuses eroding the anterior fossa; large extra-axial air collection compressing left frontal lobe. MRI: subcortical edema.	Subdural (extra-axial)	Endonasal transethmoidal endoscopic osteoma resection with multilayer skull-base and dural closure; pneumocephalus resolved, and patient was asymptomatic at 1 year.
Solavera et al. (2019) [26]	44/F: frontal headaches and sneezing-triggered clonic right arm movements generalizing to tonic–clonic seizure; prior uncomplicated frontal sinusitis.	CT/MRI: intraparenchymal pneumocephalus in the right hemisphere and a 2.4 × 2.1 × 1.8 cm anterior ethmoid osteoma eroding the lamina cribrosa.	Intraparenchymal (right frontal)	Endoscopic Draf III resection of osteoma with skull-base repair using septal mucoperichondrial graft plus Tissucol^®^/Surgicel^®^; pneumocephalus allowed to resolve spontaneously, discharged 72 h post-op without complications.
Brown & Vahidassr (2018) [27]	84/M: months of recurrent falls and unsteadiness, then 3 d of left arm/leg weakness; otherwise well.	CT: 9 cm right frontal intraparenchymal pneumatocoele with dystrophic calcification of cribriform plate and sinuses; MRI: small acute callosal ischemia.	Intraparenchymal (right frontal)	Managed conservatively with observation and rehabilitation; left-sided weakness resolved by 12 wk and remained well.

“Intraparenchymal” and “subdural” denote air collections within brain tissue or in the subdural space, respectively; “cisternal” indicates subarachnoid/cisternal accumulation. Draf III: endoscopic procedure. POD: postoperative day. CT, computed tomography; MRI, magnetic resonance imaging.

**Table 2:** Summary of reported cases of mucocele formation associated with sinonasal osteomas.

Case	Age (Years), Sex, and Presentation	Key Imaging Findings	Management and Outcome
Sakamoto et al., 2011 [4]	70/M: Generalized seizure	Frontal sinus osteoma with intracranial cystic lesion extending into the frontal lobe on CT/MRI	Frontal craniotomy with excision of osteoma and cyst, dural repair
Ali et al., 2021 [30]	35/M: Cognitive decline	Intradural mucocele and hydrocephalus due to giant frontal osteoma	VP shunt and left frontal craniotomy; complete excision of osteoma and mucocele; postoperative antibiotics for meningitis
Gezici et al. 2004 [32]	29/F: progressive right frontal proptosis, headaches, and tonic–clonic seizure	Hyperdense osteoma in frontal/ethmoid/maxillary sinuses with intracranial mucocele compressing frontal lobes; rim enhancement and edema on MRI	Frontobasal craniotomy: complete excision of osteoma and mucocele, sinus packing, dural repair, lumbar drainage → CSF leak resolved
Licci et al., 2018 [28]	49/M: Frontal lobe syndrome, pneumocephalus	Frontoethmoidal osteoma with intradural mucocele extension and pneumocephalus	Surgical resection and duraplasty → clinical improvement
Nabeshima et al., 2003 [31]	54/M: Generalized tonic seizures	Frontal sinus osteoma with intracranial cystic lesion compressing frontal lobe (CT/MRI)	Frontobasal craniotomy with excision, sinus obliteration and dural repair → uneventful recovery
Benali et al., 2023 [33]	49/F: Headache, periorbital swelling, ptosis	Frontoethmoidal osteoma with intracranial mucocele extension	Frontal craniotomy with complete excision
Samuel et al., 2024 [34]	63/F: Proptosis, frontal pain, CSF leak	Skull base proximity and mucocele	Combined open-endoscopic approach, CSF repair with fascia lata → full recovery

CT: computed tomography; MRI: magnetic resonance imaging; CSF: cerebrospinal fluid; VP shunt: ventriculoperitoneal shunt.

**Table 3:** Summary of infectious complications associated with sinonasal osteomas.

Case	Age (Years), Sex, and Presentation	Key Imaging Findings	Management and Outcome
Summers et al., 2001 [37]	51/M: headache, seizure, lethargy, right-sided weakness	Left frontal osteoma with adjacent ring-enhancing cerebral abscess	Craniotomy, abscess drainage, osteoma resection, IV antibiotics → full recovery
Kolcun et al., 2019 [6]	38/M: long-standing seizures, then meningitis	Giant frontal osteoma with dural defect and enhancing parenchymal lesion	Joint craniotomy and sinus surgery → resolution
Benzagmout et al., 2020 [38]	34/M: seizures, hemiparesis, fever	Giant frontoethmoidal osteoma with right frontoparietal subdural empyema	Craniotomy, empyema evacuation, osteoma resection, IV ceftriaxone/metronidazole → full recovery
Ramakrishna et al., 2014 [13]	71/M: fever, confusion, proptosis	Recurrent osteoma with mucopyocele and intracranial abscess (M. catarrhalis)	Preop embolization, bicoronal craniotomy, osteoma removal → resolution
Gezici et al. 2004 [32]	53/M: headache, confusion, fever, seizures with CSF staphylococcal meningitis and rhinorrhea	Dural breach by frontobasal osteoma leading to CSF leak, pneumocephalus, and bacterial meningitis	Frontobasal craniotomy: osteoma and mucocele removal, sinus packing, dural repair, antibiotics → full neurological recovery
Öztürk et al., 2020 [36]	15/M: orbital swelling, frontal pain → Pott’s puffy tumor	Frontal osteoma with anterior wall erosion and subperiosteal abscess	IV antibiotics, later osteoma resection → complete recovery
Bagheri et al., 2021 [35]	30/F: orbital cellulitis, proptosis, fever	Ethmoidal osteoma extending into orbit; sinus opacification	IV antibiotics, endoscopic and open resection → full recovery

IV: intravenous.

**Table 4:** Summary of orbital complications associated with sinonasal osteomas.

Case	Age (Years), Sex, and Presentation	Key Imaging Findings	Management and Outcome
Satyarthee et al., 2015 [40]	14/M: Progressive unilateral visual loss, optic atrophy, and facial deformity	Giant sphenoethmoidal osteoma with orbital extension and optic nerve compression	Surgical decompression and tumor excision; visual improvement reported
Handa et al., 2024 [7]	28/M: Progressive globe displacement and facial asymmetry	Giant frontoethmoidal osteoma causing significant orbital displacement	Combined surgical approach; good cosmetic and functional outcome
Ata et al., 2017 [41]	Adult patient: Ptosis and orbital swelling	Large frontoorbital osteoma causing localized orbital compression	Surgical excision; complete resolution of ptosis
Dari & Gdey, 2024 [22]	30/F: Hemiparesis, orbital symptoms	45 × 42 mm osteoma compressing orbit and frontal lobe	Bicoronal approach with Lynch extension → complete resection

**Table 5:** Reported cases of sinonasal osteomas in rare anatomical locations.

Case	Age (Years), Sex, and Presentation	Key Imaging Findings	Management and Outcome
Gulsen et al., 2019 [43]	15/M: intractable frontal headache	Hyperdense lesion arising within the middle concha bullosa	Endoscopic removal; complete resolution of headache
Erdogan et al., 2017 [44]	46/F: chronic frontal headache	Osteoma located within a pneumatized septal air cell	Endoscopic removal; symptom resolution at 6-month follow-up
Kumar et al., 2010 [45]	54/F: nasal obstruction, facial pain, and headache	Hyperdense mass occupying the inferior turbinate and nasal cavity	Endoscopic excision using microdrill; complete symptom resolution
Yazici et al., 2014 [46]	21/M: progressive facial swelling	2 × 2 cm ossified lesion of the nasal bone	Endonasal endoscopic excision with MEDPOR reconstruction; no recurrence
Hwang et al., 2019 [47]	34/F: cosmetic nasal deformity and facial swelling	0.7 × 0.5 cm radiopaque lesion arising from the nasal bone	Excision via open rhinoplasty; no recurrence and preserved nasal symmetry
Strek et al., 2005 [48]	19/F: progressive headache	Large sphenoid sinus osteoma identified on CT imaging	Endoscopic resection; complete symptom resolution

## Data Availability

No new data were created or analyzed in this study.

## Abbreviations

The following abbreviations are used in this manuscript:

CT	Computed tomography
MRI	Magnetic resonance imaging
CSF	Cerebrospinal fluid
VP	Ventriculoperitoneal
ESS	Endoscopic sinus surgery
PEEK	Polyetheretherketone
POD	Postoperative day
IV	Intravenous
Draf III	Endoscopic modified Lothrop procedure
ICP	Intracranial pressure
